# Unraveling the causal role of immune cells in gastrointestinal tract cancers: insights from a Mendelian randomization study

**DOI:** 10.3389/fimmu.2024.1343512

**Published:** 2024-03-12

**Authors:** Yu-xiang Wang, Chao-ping Zhou, Da-tian Wang, Jun Ma, Xue-hu Sun, Yao Wang, Ya-ming Zhang

**Affiliations:** ^1^ Department of General Surgery, Anqing Municipal Hospital, Anqing, Anhui, China; ^2^ Department of Emergency Surgery, The First Affiliated Hospital of Anhui Medical University, Hefei, Anhui, China; ^3^ Department of Digestive Endoscopy, The First Affiliated Hospital with Nanjing Medical University, Nanjing, Jiangsu, China

**Keywords:** tumor microenvironment, immunology, cancer, B cells, gastric cancer

## Abstract

**Background:**

Despite early attempts, the relationship between immune characteristics and gastrointestinal tract cancers remains incompletely elucidated. Hence, rigorous and further investigations in this domain hold significant clinical relevance for the development of novel potential immunotherapeutic targets.

**Methods:**

We conducted a two-sample Mendelian randomization (MR) analysis using the tools available in the “TwoSampleMR” R package. The GWAS data for these 731 immune traits were sourced from the GWAS Catalog database. Concurrently, data on gastrointestinal tract cancers, encompassing malignant tumors in the esophagus, stomach, small intestine, colon, and rectum, were extracted from the FinnGen database. The immune traits subjected to MR analysis predominantly fall into four categories: median fluorescence intensities (MFI), relative cell (RC), absolute cell (AC), and morphological parameters (MP). To ensure the reliability of our findings, sensitivity analyses were implemented to address robustness, account for heterogeneity, and alleviate the impact of horizontal pleiotropy.

**Results:**

A total of 78 immune traits causally linked to gastrointestinal tract cancers were identified, encompassing esophageal cancer (12 traits), gastric cancer (13 traits), small intestine cancer (22 traits), colon cancer (12 traits), and rectal cancer (19 traits). Additionally, 60 immune traits were recognized as protective factors associated with gastrointestinal tract cancers, distributed across esophageal cancer (14 traits), gastric cancer (16 traits), small intestine cancer (7 traits), colon cancer (14 traits), and rectal cancer (9 traits). Furthermore, it was observed that seven immune traits are causally related to gastrointestinal tract cancers in at least two locations. These traits include “CCR2 on CD14- CD16+ monocyte,” “CD19 on IgD+ CD38-,” “CD19 on IgD+ CD38- naive,” “CD25hi CD45RA+ CD4 not Treg AC,” “CD27 on unsw mem,” “CD28 on CD39+ activated Treg,” and “CD45 on CD4+.”

**Conclusion:**

This study elucidates a causal link between immune cells and gastrointestinal tract cancers at various sites through genetic investigation. The findings of this research open up new perspectives and resources for exploring tumor prevention strategies and immunotherapeutic targets.

## 1 Introduction

Gastrointestinal tract cancers, comprising malignant tumors in the esophagus, stomach, small intestine, colon, and rectum, represent ubiquitous lethal malignancies in human beings (1). Existing epidemiological evidence indicates a significant increase in the incidence of gastrointestinal tract cancers, particularly colorectal cancer and stomach cancer, among the youth population within the past three decades (1–3). The majority of these early-onset cases lack significant genetic or familial backgrounds, implicating potential critical roles of certain lifestyle, nutritional, metabolic, and environmental factors in cancer development (1). Therefore, an in-depth exploration of the associated risk factors of gastrointestinal tract cancers is of paramount importance for the prevention of gastrointestinal tract cancers, as well as for the development of potential anticancer agents.

In recent decades, growing body of evidence has demonstrated the crucial linkage between immune cells and the onset and progression of gastrointestinal tract cancers (4–7). For example, single-cell sequencing data has revealed that T lymphocytes and natural killer cells with exhaustion, regulatory T cells, alternatively activated macrophages, and tolerant dendritic cells dominate the tumor microenvironment of esophageal cancer (8). Moreover, it has been reported that tumor-associated macrophages (TAMs) polarize towards pro-inflammatory phenotype and induce gastric cancer cell apoptosis through IL6R-JAK-IL24 pathway, upon STING knockdown or 2’3’-c-GAMPSTING activation (4). The immune response of CD8+ T cells in regulating colorectal cancer has a significant impact on tumoral proliferation and metastasis (9). In rectal cancer, not only is the high intra-tumoral CD8+ cell density associated with improved overall survival, but also the high density of PD-1+ and CD8+ immune cells before treatment is significantly correlated with favorable response to neoadjuvant chemoradiotherapy (CRT) and improved recurrence-free survival (10). Moreover, it is noteworthy that immunotherapy has emerged as a potent clinical strategy for treating cancers (11). The number of approved immunotherapeutic drugs has been increasing, and many treatment modalities are currently under clinical and pre-clinical development (12). In summary, the incidence, progression, and clinical drug development of gastrointestinal tract cancers are closely related to immune cells. However, most studies only establish the correlation between immune cells and tumoral characteristics and fail to elucidate the directionality, i.e., the causal relationship, of this correlation. Therefore, it is of utmost importance to further investigate the causal relationship between immune cells and gastrointestinal tract cancers and screen potential immune cells as targets for prevention and treatment of gastrointestinal tract cancers.

Mendelian Randomization (MR) is a data analysis technique used in epidemiological research to evaluate causal inference. It applies genetic variation as an instrumental variable (IV) to estimate the causal relationship between the exposure factor and the outcome (13). MR utilizes the first and second laws of Mendelian inheritance, which state that the parental alleles are randomly assigned to the offspring during meiosis, so the relationship between the genes and the outcome is not affected by common confounding factors such as postnatal environment, socioeconomic status, and behavior. Therefore, the causal relationship inferred by MR has a reasonable temporal sequence (14).

In this study, we aimed to employ MR analysis to comprehensively investigate the causal relationship between different immune traits and gastrointestinal tract cancers. These findings provide resources and new insights for exploring potential targets for the prevention and treatment of gastrointestinal tract cancers.

## 2 Materials and methods

### 2.1 Study design

**Figure 1:**
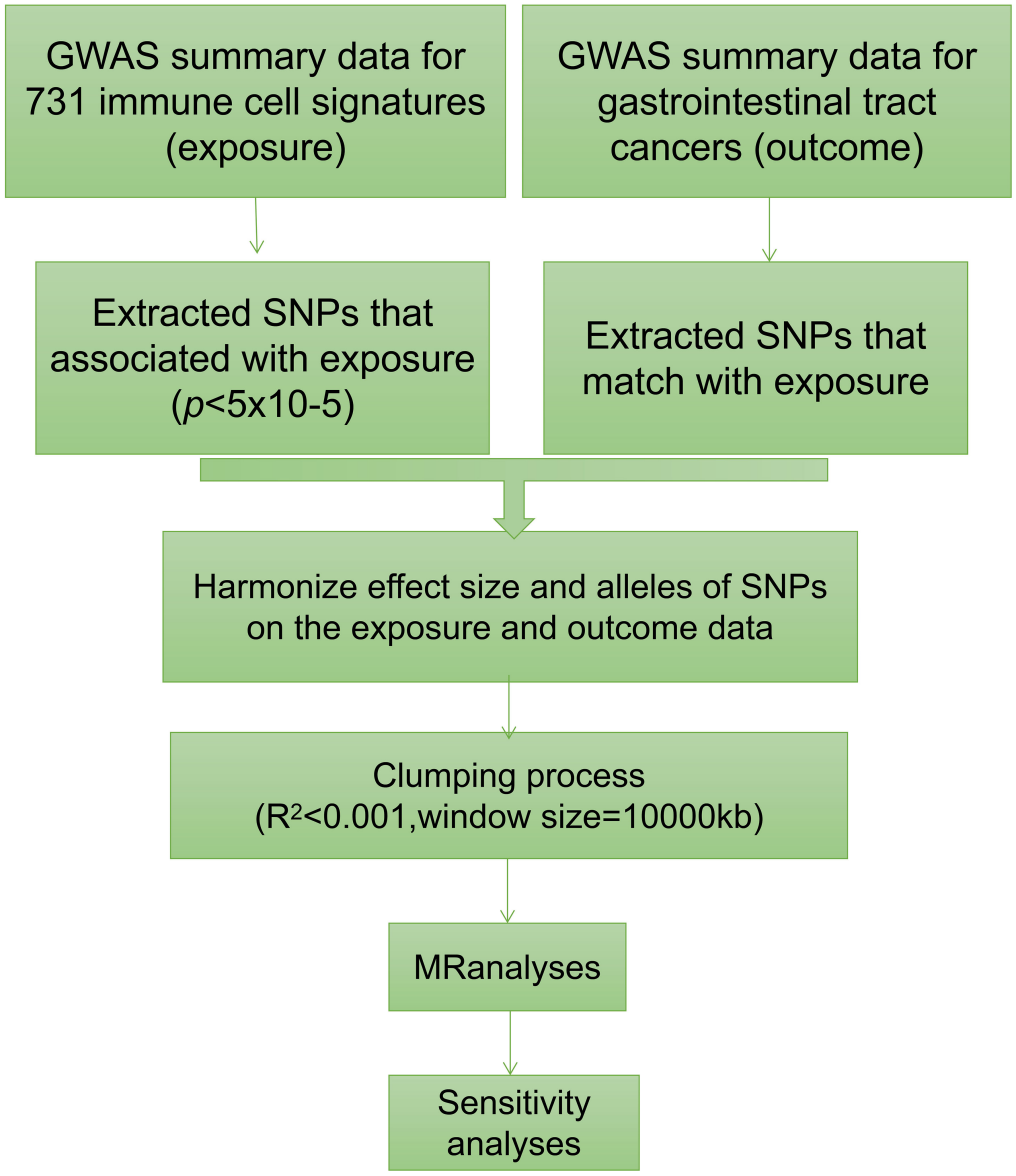
The flowchart graph of this study.

We conducted a comprehensive assessment to investigate the causal relationship between 731 immune traits, classified into seven groups (refer to **Supplementary Table 1**: B cell, cDC, TBNK, Treg, Myeloid cell, Maturation stages of T cell, and Monocyte), and gastrointestinal tract cancers using a two-sample MR approach (**Figure 1**) (15). The studied gastrointestinal tract cancers include those affecting the esophagus, stomach, small intestine, colon, and rectum. In MR analysis, genetic variants act as proxies for risk factors, necessitating IVs to adhere to three critical assumptions for valid causal inference: (1) a direct association exists between genetic variation and the exposure of interest; (2) genetic variation remains unrelated to potential confounding variables lying between the exposure and outcome; and (3) genetic variation does not influence the outcome through pathways distinct from the exposure (16–18).

### 2.2 Genome-wide association study data sources

The GWAS summary statistics for total of five types of gastrointestinal tract cancers were sourced from the FinnGen database (19). Detailed information, including the number of patients and controls involved for each type of gastrointestinal tract cancer, can be found in the **Supplementary Table 2**. The URL for downloading the data pertaining to each type of gastrointestinal tract cancer is also incorporated in **Supplementary Table 2**. The diagnoses of all patients were made according to the ICD10 code. Additionally, the control group included individuals without any history of cancer.

We systematically retrieved immune trait-related signatures from the GWAS Catalog database, aiming for a comprehensive inclusion of relevant data. The final compilation encompassed a total of 731 immune traits, intricately representing diverse subsets of human immune cells (15). These signatures include absolute cell (AC) counts (n=118), median fluorescence intensities (MFI) reflecting surface antigen levels (n=389), morphological parameters (MP) (n=32), and relative cell (RC) counts (n=192).

The use of MFI, AC, and RC as quantitative or intensity units spanned various immune cell populations, including B cells, CDCs, mature stages of T cells, monocytes, myeloid cells, TBNK (T cells, B cells, natural killer cells), and Treg panels. Morphological parameters (MP) were additionally employed to represent indicators of CDC and TBNK panels.

The genetic data associated with these immune traits were sourced from 3,757 European individuals, ensuring no overlap with the GWAS data for gastrointestinal tract cancers. In the GWAS dataset, each sample underwent scrutiny for approximately 22 million single nucleotide polymorphisms (SNPs). Notably, the associations between these SNPs and the immune traits were meticulously examined, with consideration given to covariates such as sex, age, and age^2^ during the analysis.

### 2.3 Selection of instrumental variables

Aligned with current research standards, the threshold for the significance of IVs linked to each immune trait was set at 1×10-5 (20–23). To refine the selection of SNPs, we implemented a clumping procedure, applying a linkage disequilibrium (LD) r2 threshold of less than 0.001 within a 10,000 kb distance (24–26). Subsequently, we computed F-statistics for each IV to assess their strength and mitigate potential instrumental bias. IVs with F-statistics below 10 were excluded from the analysis. This rigorous process resulted in the identification of a variable range, spanning 3 to 753 independent IVs associated with immunophenotypes, as detailed in **Supplementary Table 3**.

### 2.4 Statistical analysis

All computational analyses were performed using R 4.2.1. To assess the causal relationships between 731 immune trait-related signatures and gastrointestinal tract cancers, a comprehensive set of MR approaches, including Inverse Variance Weighting (IVW), MR Egger, Weighted Median, Simple Mode, and Weighted Mode, were executed utilizing the “TwoSampleMR” R package (version 0.5.7) (27–29).

To evaluate the presence of heterogeneity among the selected IVs, Cochran’s Q statistic was applied (23). To mitigate the influence of horizontal pleiotropy, the widely recognized MR-Egger method was utilized, and the significance of its intercept term indicated potential horizontal pleiotropy (16).

To further guard against the impact of horizontal pleiotropy and the presence of potential outliers, we implemented the robust MR-Pleiotropy Residual Sum and Outlier (MR-PRESSO) method, integrated into the MR-PRESSO package (15, 23). Additionally, scatter plots and funnel plots were used for visual inspection of the data. Scatter plots confirmed resistance to the influence of outliers, while funnel plots illustrated the robustness of correlations and the absence of significant heterogeneity in the results (29).

In this study, we employed immune trait-related signatures as the exposure, and five different types of gastrointestinal tract cancer as the outcome, for conducting MR analysis. The IVW method was utilized as the primary analysis method in our study. We considered a causal relationship between the exposure of immune trait and the outcome of gastrointestinal tract cancer, when the *p*-value derived from the IVW method was smaller than 0.05 and the odds ratio (OR) estimates obtained from other MR methods such as MR Egger, Weighted Median, Simple Mode, and Weighted Mode were greater or smaller than 1 or 0, respectively.

## 3 Results

### 3.1 Exploration of the causal effect of immune traits on esophagus cancer

**Figure 2:**
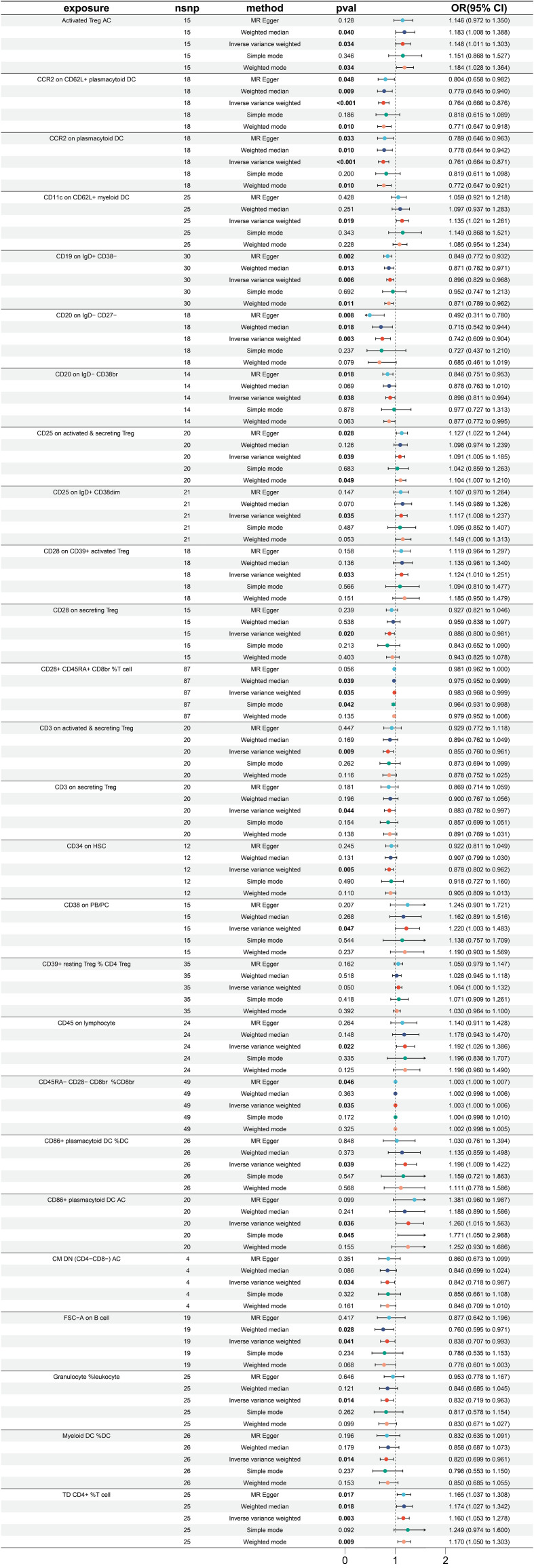
Forest plot for MR analysis with esophagus cancer as the outcome.

A total of 26 immune traits were found to have a significant causal relationship with the occurrence of esophageal cancer. According to the MR analysis based on the IVW method, 14 immune traits were determined to have a protective effect against the onset of esophageal cancer, while the remaining 12 immune traits were identified as risk factors of esophageal cancer (**Figure 2**). The immune traits with *p-*values less than 0.05 obtained by the IVW method are shown in **Supplementary Figure 1A**. The top three immune traits with the smallest OR values among the protective factors against esophageal cancer were “CD20 on IgD- CD27-” (IVW *p*=0.003; OR=0.742, 95%CI [0.609-0.904]), “CCR2 on plasmacytoid DC” (IVW *p<*0.001; OR=0.761, 95%CI [0.664-0.871]), and “CCR2 on CD62L+ plasmacytoid DC” (IVW *p<*0.001; OR=0.764, 95%CI [0.666-0.876]). The IVW and MR Egger tests were employed to assess the heterogeneity in the identified protective factors. The Q *p*-values of the three protective factors obtained from the IVW heterogeneity test were 0.582, 0.940, and 0.838, respectively, while the Q *p*-values obtained from the MR Egger test were 0.781, 0.922, and 0.817 (**Supplementary Figure 2A**).

On the other hand, the top three immune traits with the largest OR values among the risk factors for esophageal cancer were “CD86+ plasmacytoid DC %DC” (IVW *p =*0.039; OR=1.198, 95%CI [1.009-1.422]), “CD38 on PB/PC” (IVW *p =*0.047; OR=1.220, 95%CI [1.003-1.483]), and “CD86+ plasmacytoid DC AC” (IVW *p =*0.036; OR=1.260, 95%CI [1.015-1.563]). The Q *p*-values of these three risk factors obtained from the IVW heterogeneity test were 0.604, 0.566, and 0.200, respectively, while the Q *p*-values obtained from the MR Egger test were 0.630, 0.489, and 0.175 (**Supplementary Figure 2A**).

Furthermore, there was no substantial pleiotropy observed, as indicated by the Egger intercept (**Supplementary Figure 2B**). In leave-one-out analyses, altering a single SNP did not alter the direction of the results (**Supplementary Figure 2C**).

Worth noting is that there exist two protective variables, namely, the “CCR2 on plasmacytoid DC” and “CCR2 on CD62L+ plasmacytoid DC”. Albeit having yielded non-significant *p* values under the “Simple mode” analytical approach, all other MR analytical techniques have produced *p* values of less than 0.05, suggestive of significant calculated results of the aforementioned protective factors (**Figure 2**).

### 3.2 Exploration of the causal effect of immune traits on stomach cancer

**Figure 3:**
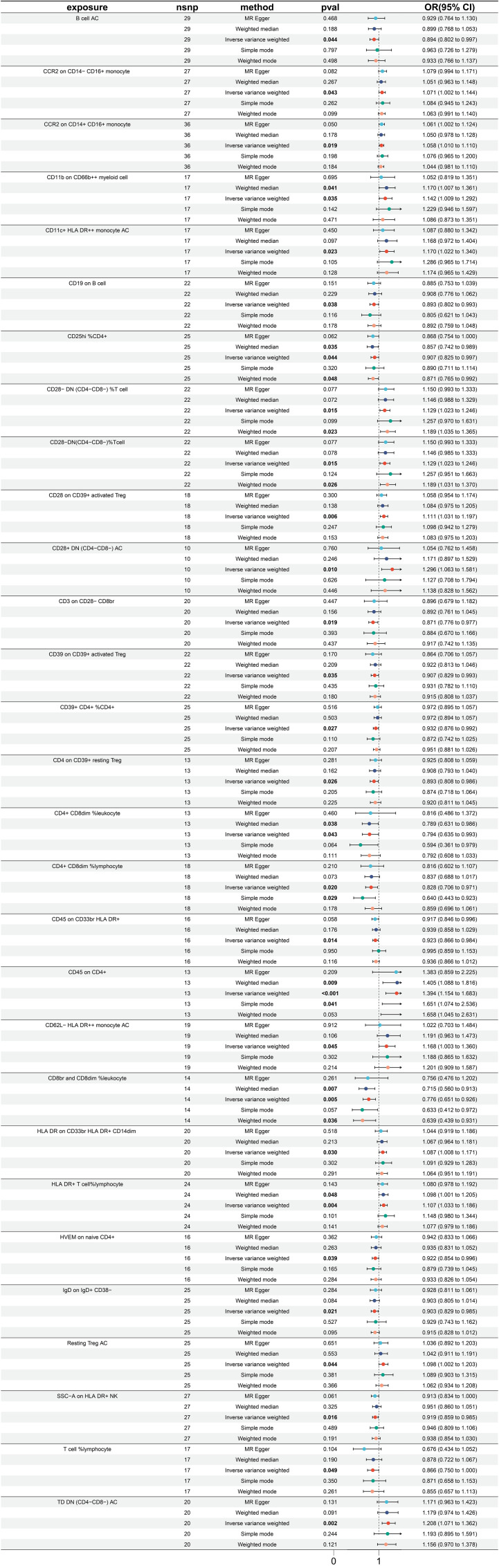
Forest plot for MR analysis with stomach cancer as the outcome.

In totality, 29 immune traits were detected to exhibit a significant causal relationship with gastric cancer. Upon conducting MR analysis using the IVW approach, it was determined that 16 immune traits serve a protective role in gastric cancer incidence, whereas the remaining 13 immune traits were identified as risk factors for gastric cancer (refer to **Figure 3**). The immune traits with *p*-values less than 0.05 obtained by the IVW method are shown in **Supplementary Figure 1B**. Referring to the 16 identified protective factors, the three immune traits with the smallest odds ratios, denoted as “CD8br and CD8dim %leukocyte” (IVW *p*=0.004; OR=0.766, 95%CI [0.651 ~ 0.926]), “CD4+ CD8dim %leukocyte” (IVW *p*=0.043; OR=0.794, 95%CI [0.635 ~ 0.993]), and “CD4+ CD8dim %lymphocyte” (IVW *p*=0.020; OR=0.828, 95%CI [0.706 ~ 0.971]), were assessed for heterogeneity through the application of both IVW and MR Egger tests. The IVW heterogeneity tests produced Q *p*-values of 0.804, 0.030, and 0.086, and the MR Egger heterogeneity tests yielded values of 0.740, 0.020, and 0.064, respectively (**Supplementary Figure 3A**).

Of the identified immune traits contributing to gastric cancer risk, the three exhibiting the highest OR were “CD45 on CD4+” (IVW p <0.001; OR=1.394, 95%CI [1.154 ~ 1.683]), “CD28+ DN (CD4-CD8-) AC” (IVW p =0.010; OR=1.296, 95%CI [1.063 ~ 1.581]), and “TD DN (CD4-CD8-) AC” (IVW p =0.002; OR=1.208, 95%CI [1.071 ~ 1.362]). The assessment of heterogeneity of these three identified risk factors through the utilization of IVW tests resulted in Q *p* values of 0.242, 0.522, and 0.404, while the MR Egger tests yielded values of 0.183, 0.690, and 0.353, respectively (**Supplementary Figure 3A**). Furthermore, no significant pleiotropy was observed as illustrated by the Egger intercept (**Supplementary Figure 3B**) and the leave-one-out analysis demonstrated that the results were not influenced by the inclusion of any single SNP (**Supplementary Figure 3C**).

### 3.3 Exploration of the causal effect of immune traits on cancer of small intestine

**Figure 4:**
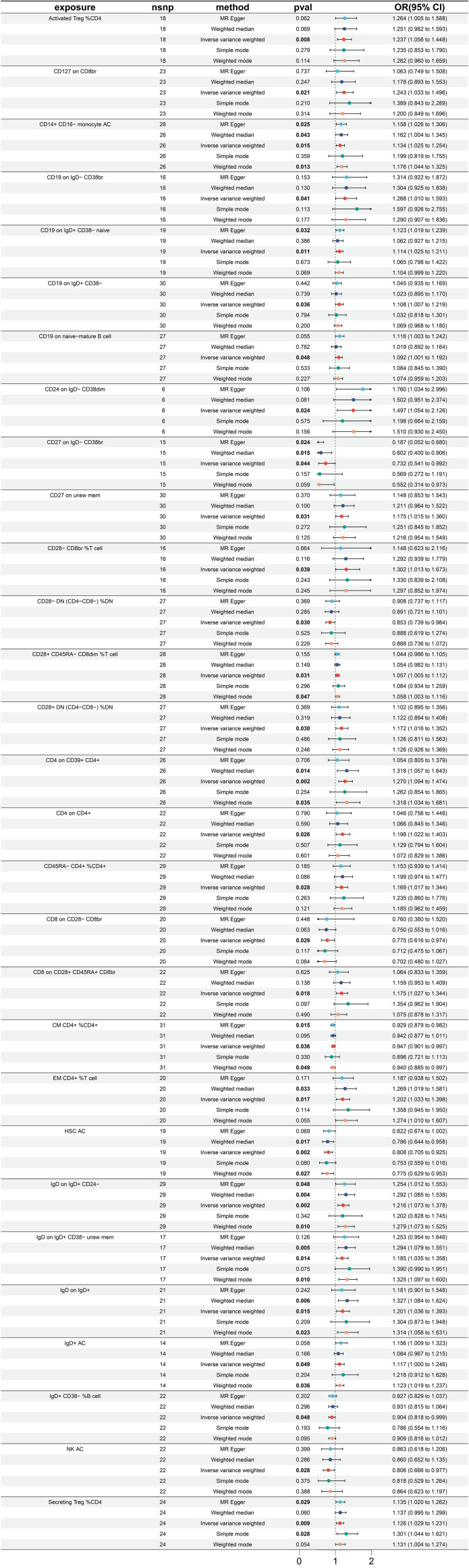
Forest plot for MR analysis with cancer of small intestine as the outcome.

Utilizing MR analysis, a total of 29 immune traits were identified to have significant causal relationships with small intestine cancer, with the majority being risk factors, while only 7 immune traits were identified to exhibit protective properties (**Figure 4**). The immune traits with *p*-values less than 0.05 obtained by the IVW method are shown in **Supplementary Figure 1C**. The immune traits exhibiting the three smallest ORs as protective factors were “CD27 on IgD- CD38br” (IVW p=0.044; OR=0.732, 95%CI [0.541 ~ 0.992]), “CD8 on CD28- CD8br” (IVW p=0.029; OR=0.775, 95%CI [0.616 ~ 0.973]), and “NK AC” (IVW p=0.028; OR=0.806, 95%CI [0.666 ~ 0.977]). The heterogeneity of the aforementioned protective factors were evaluated through the employment of IVW and MR Egger tests, resulting in Q *p* values of 0.331, 0.898, and 0.499 and 0.594, 0.863, and 0.451, respectively (**Supplementary Figure 4A**). Within the remaining immune traits associated with risk, the three with the highest ORs were “CD24 on IgD- CD38dim” (IVW p =0.024; OR=1.497, 95%CI [1.054 ~ 2.126]), “CD28- CD8br %T cell” (IVW p =0.039; OR=1.302, 95%CI [1.013 ~ 1.673]), and “CD4 on CD39+ CD4+” (IVW p =0.002; OR=1.270, 95%CI[1.094 ~ 1.474]). The heterogeneity of these three identified risk factors were assessed through IVW testing, resulting in Q *p* values of 0.908, 0.997, and 0.585, while the MR Egger test yielded values of 0.922, 0.995, and 0.686, respectively (**Supplementary Figure 4A**). Furthermore, no significant pleiotropy was observed, as demonstrated by the Egger intercept (**Supplementary Figure 4B**) and the results were not influenced by the inclusion of any single SNP, as demonstrated by the leave-one-out analysis (**Supplementary Figure 4C**).

### 3.4 Exploration of the causal effect of immune traits on colon cancer

**Figure 5:**
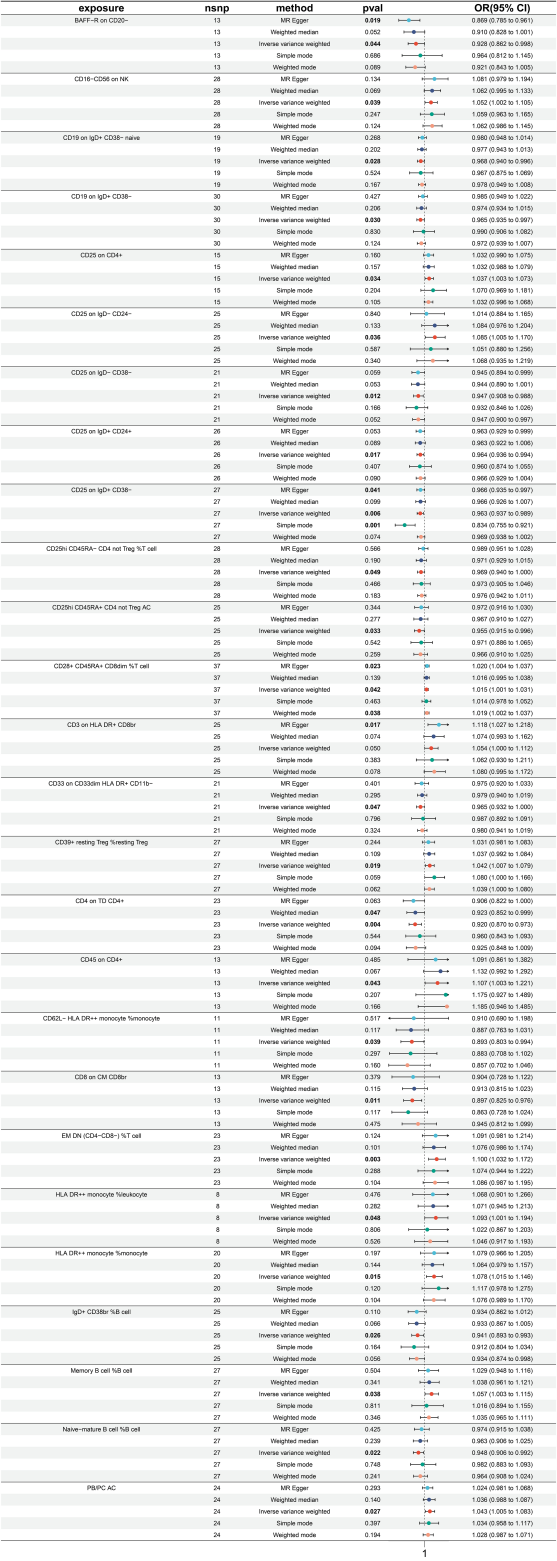
Forest plot for MR analysis with colon cancer as the outcome.

A total of 26 immune traits have been identified to exhibit significant causal relationships with colon cancer, with 12 being risk factors and 14 being protective factors (**Figure 5**). The immune traits with *p*-values less than 0.05 obtained by the IVW method are shown in **Supplementary Figure 1D**. The top 3 immune traits with the smallest ORs as protective factors were “CD62L- HLA DR++ monocyte %monocyte” (IVW p=0.039; OR=0.893, 95%CI [0.603 ~ 0.994]), “CD8 on CM CD8br” (IVW p=0.011; OR=0.897, 95%CI [0.825 ~ 0.976]), and “CD4 on TD CD4+” (IVW p=0.004; OR=0.920, 95%CI [0.870 ~ 0.973]). The heterogeneity of the aforementioned protective factors were evaluated through the employment of IVW and MR Egger tests, resulting in Q *p* values of 0.882 (IVW), 0.892 (IVW), and 0.429 (IVW), and 0.824 (Egger), 0.842 (Egger), and 0.378 (Egger), respectively (**Supplementary Figure 5A**). Within the immune traits associated with colon cancer risk, the three with the highest ORs were “CD45 on CD4+” (IVW p=0.043; OR=1.107, 95%CI [1.003 ~ 1.221]), “EM DN (CD4-CD8-) %T cell” (IVW p=0.003; OR=1.100, 95%CI [1.032 ~ 1.172]), and “HLA DR++ monocyte %leukocyte” (IVW p=0.048; OR=1.093, 95%CI [1.001 ~ 1.194]). The heterogeneity of these three identified risk factors were assessed through IVW testing, resulting in Q *p* values of 0.879, 0.598, and 0.628, while the MR Egger test yielded values of 0.826, 0.539, and 0.522, respectively (**Supplementary Figure 5A**). Additionally, the Egger intercept was not significant, as indicated in **Supplementary Figure 4B**, and the leave-one-out analysis suggested that the results were not influenced by any single SNP (**Supplementary Figure 5C**).

### 3.5 Exploration of the causal effect of immune traits on rectal cancer

**Figure 6:**
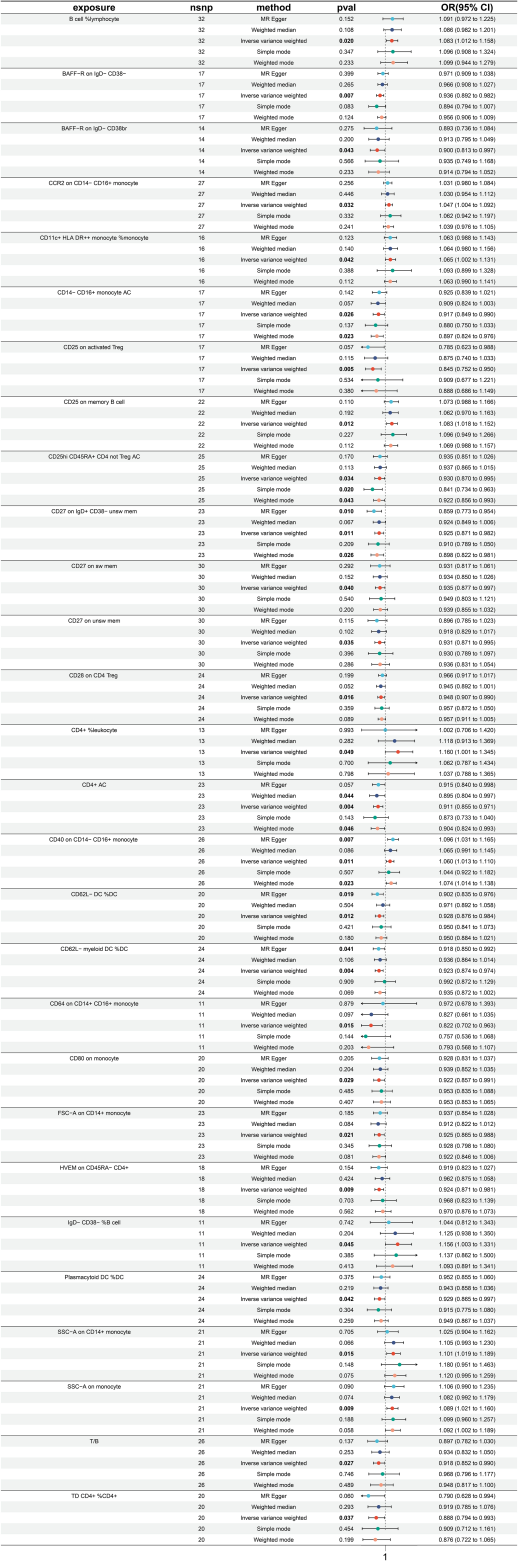
Forest plot for MR analysis with rectal cancer as the outcome.

A significant causal relationship between 28 immune traits and rectal cancer has been discovered, with 19 identified as risk factors and only 9 as protective factors (**Figure 6**). The immune traits with *p*-values less than 0.05 obtained by the IVW method are shown in **Supplementary Figure 1D**. The top three protective factors with the smallest ORs were identified as “CD64 on CD14+ CD16+ monocyte” (IVW p=0.0154; OR=0.822, 95%CI[0.702 ~ 0.963]), “CD25 on activated Treg” (IVW p=0.005; OR=0.845, 95%CI[0.752 ~ 0.951]), and “TD CD4+ %CD4+” (IVW p=0.037; OR=0.888, 95%ci[0.794 ~ 0.993]). The heterogeneity of these three factors was evaluated by IVW test, with corresponding Q *p* values of 0.491, 0.544, and 0.301, and by Egger test, with Q *p* values of 0.492, 0.511, and 0.320 respectively (**Supplementary Figure 6A**). On the other hand, the top three immune traits with the highest ORs were “CD4+ %leukocyte” (IVW p=0.049; OR=1.160, 95%CI[1.001 ~ 1.345]), “IgD- CD38- %B cell” (IVW p=0.045; OR=1.156, 95%CI[1.003 ~ 1.331]), and “SSC-A on CD14+ monocyte” (IVW p=0.015; OR=1.101, 95%ci[1.019 ~ 1.189]). The heterogeneity of these identified risk factors was also evaluated by IVW test, with corresponding Q *p* values of 0.749, 0.394, and 0.632, and MR Egger test, with values of 0.747, 0.386, and 0.701 (**Supplementary Figure 6A**). Furthermore, **Supplementary Figure 6B** displays Egger intercepts, indicating the absence of significant pleiotropy, while leave-one-out analysis suggests the results are not influenced by the inclusion of any single SNP (**Supplementary Figure 6C**).

### 3.6 Exploring immune traits causally related to gastrointestinal tract cancers of different sites

**Figure 7:**
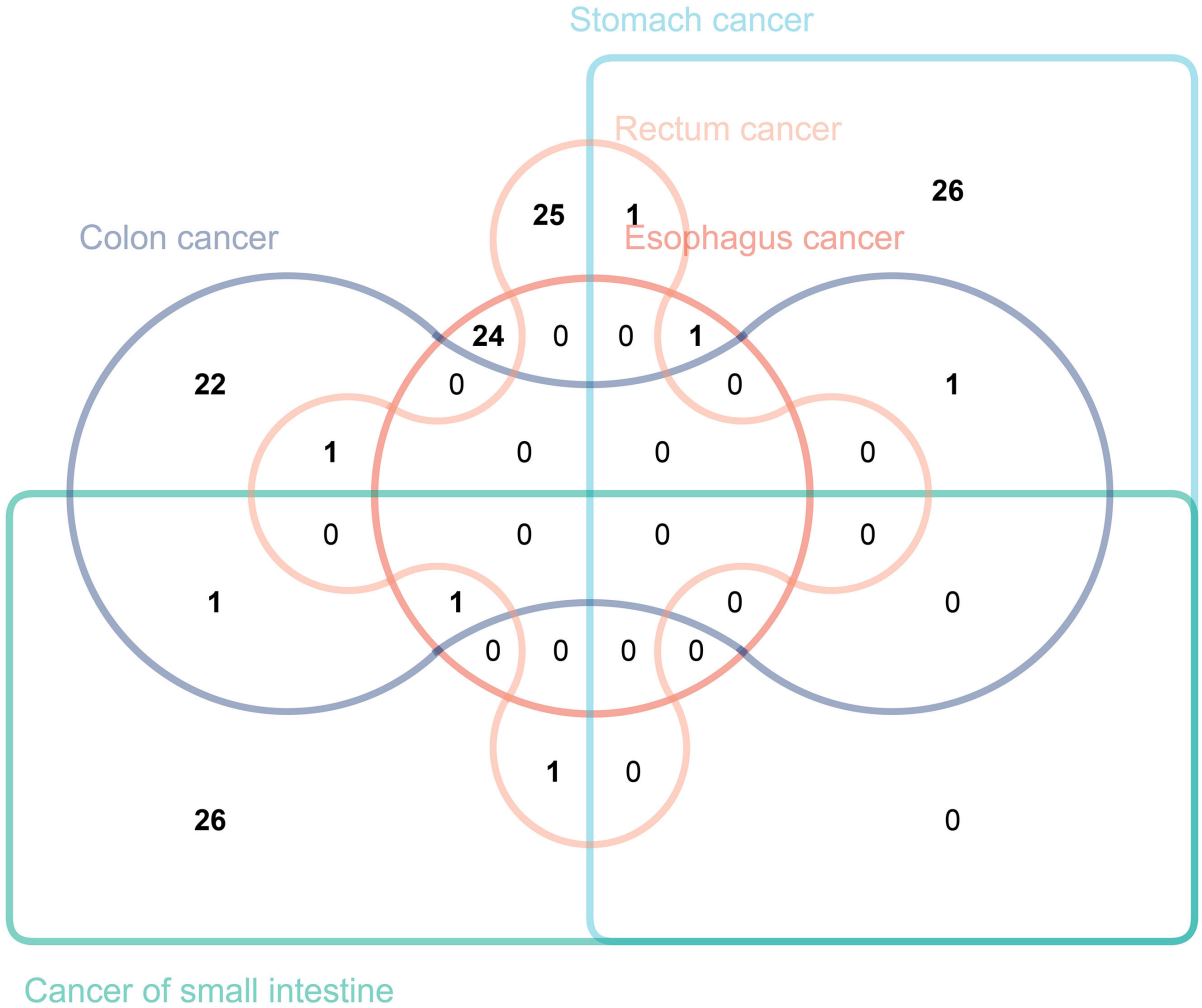
The Venn diagram illustrating the number of immune traits causally associated with various gastrointestinal tract tumors.

In this study, we intersected immune traits causally related to gastrointestinal tract cancers of different sites with those previously identified (**Figure 7**). While no immune traits were found to be causally related to the occurrence of all five types of tumors, this study has identified seven immune traits as causally related to the occurrence of two or more types of tumors. Specifically, “CCR2 on CD14- CD16+ monocyte” was found to have a causal relationship with both stomach cancer and rectal cancer. “CD45 on CD4+” was identified to be causally related to both stomach cancer and colon cancer. Moreover, “CD28 on CD39+ activated Treg” was causally related to stomach cancer and esophageal cancer, while “CD19 on IgD+ CD38- naive” was found to be causally related to colon cancer and cancer of the small intestine. The study also revealed that “CD25hi CD45RA+ CD4 not Treg AC” was causally related to rectal cancer and colon cancer, and “CD27 on unsw mem” to rectal cancer and cancer of the small intestine. It is noteworthy that CD19 on IgD+ CD38- was found to be causally related to esophageal cancer, colon cancer, and cancer of the small intestine. We validated our results using the BioBank Japan (BBJ) database (https://biobankjp.org/en/index.html). Since data for esophageal cancer, gastric cancer, and colon cancer are only available in the BBJ database, we verified “CD45 on CD4+” and “CD28 on CD39+ activated Treg” only. Detailed MR analysis results can be found in **Supplementary Table 4**. The validation results from the BBJ database are consistent with our research findings.

## 4 Discussion

In our study, we conducted a comprehensive analysis of 731 distinct immune traits to identify and screen for immune traits causally associated with different gastrointestinal tract cancers. From this analysis, we identified seven distinct immune traits that were causally linked to various cancers. This research not only strengthens the existing body of knowledge regarding the essential role of immune cells in the development and progression of cancers, but also provides fundamental insights and a new perspective on the prevention and treatment of tumors, and the development of novel anti-cancer therapies.

It is noteworthy that nearly all MR analysis methodologies have suggested a protective role of plasmacytoid dendritic cells (pDCs) against esophageal cancer. Previous studies have demonstrated that pDCs represent a subtype of dendritic cells that can generate copious amounts of type I interferon (IFN-I/α) (30). Under normal conditions, TLR-activated pDCs produce potent IFN-α, thereby promoting both innate and adaptive immune responses. However, within the context of cancer, the activation response of pDCs to TLR7/9 is impaired, resulting in decreased or absent production of IFN-α, which in turn, leads to the establishment of an immunosuppressive tumor microenvironment (31). Beyond their production of IFN-α, pDCs also function as antigen-presenting cells (APCs), regulating immune responses to various antigens (32). While clinical trial outcomes of DC-based vaccines have proved disappointing, recent research has underscored the pivotal role of DC-mediated cross-priming in eliciting anti-tumor CD8 T cell immunity and modulating the anti-tumor effects of immunotherapies. Consequently, these emerging findings advocate for further advancement and refinement of DC-based vaccines, positioning them as standalone immunotherapies or in combination with other immunotherapies (33). Given pDCs’ critical role in modulating both innate and adaptive components of the immune system, they are poised to play a central role in cancer immunology.

Moreover, within the small intestine, four of five MR-based analytical methods indicate a significant increase in cancer incidence risk associated with CD14+CD16− monocytes. CD14+ CD16- monocytes, classified as classical monocytes due to their high CD14 expression and lack of CD16 expression, have been shown in previous studies to possess the capacity to secrete elevated levels of cytokines, including IL-6, CCL2, and G-CSF (34, 35). Among these cytokines, IL-6 is a major cytokine present in the tumor microenvironment and is overexpressed in almost all types of tumors. IL-6 promotes cancer progression by regulating tumor markers and multiple signaling pathways, including apoptosis, survival, proliferation, angiogenesis, invasion, and metastasis, as well as metabolism (36, 37). CCL2 is able to promote cancer cell growth and proliferation through various mechanisms. By interacting with CCR2, CCL2 facilitates cancer cell migration and recruits immunosuppressive cells to the tumor microenvironment, thereby promoting cancer development (38). Numerous preclinical investigations have elucidated the tumor-promoting impact of granulocyte colony-stimulating factor (G-CSF), predominantly orchestrated by neutrophils and MDSCs, the primary subset expressing G-CSF receptors. In the presence of tumor-derived or exogenous G-CSF, these myeloid cell populations typically demonstrate a T-cell suppressive phenotype (39). Consequently, the high levels of cytokines secreted by CD14+ CD16- monocytes partly explain the tumor risks associated with these cells.

The MR analysis in this study suggests that CD4+ T cells serve as a protective factor against rectum cancer. Previous investigation have revealed that CD4+ T cells not only express key molecules associated with cytolysis (such as Granzymes [GZM] and Perforin [PRF1]) but also possess direct cytotoxicity, forming the basis for their protective immunity, including in cancer (40). CD4+ T cells can engage tumor cells through various mechanisms, either by directly eliminating tumor cells via cytolysis or indirectly by modulating the tumor microenvironment (41). Additionally, in secondary lymphoid organs, CD4+ T cells amplify the intensity and quality of B cell and Cytotoxic T lymphocytes (CTLs) responses. Antigen-specific interaction with CD4+ T cells enables dendritic cells (DCs) to optimize antigen presentation and deliver specific cytokine and co-stimulatory signals to CD8+ T cells, facilitating their clonal expansion and differentiation into effector or memory T cells (40, 42). CD4+ T cells assist in initiating the gene expression program of CD8+ T cells, which enhances CTL function through various molecular mechanisms, enabling them to overcome obstacles commonly encountered in anticancer immunity (43).

CD19, a CD molecule expressed by B cells, is utilized within this signature to evaluate the level of IgD+ CD38- B cells based on fluorescence intensities in “CD19 on IgD+ CD38-” signature. As a subtype of Naive B cells, IgD+ CD38- B cells are suggested, based on MR analysis, to have a causal relationship with three types of gastrointestinal tract cancers (44). Naive B cells, referring to immature B lymphocytes that have yet to experience antigenic stimulation, are included within this B cell subset (45). These cells typically reside within lymphoid tissues, the spleen, and bone marrow, with a relatively short lifespan but the potential to react to a broad spectrum of antigens. Upon encountering specific antigens within lymphoid tissues during an infection, they receive assistance from T cells for differentiation and antibody production to combat the invading pathogen (46). Therefore, Naive B cells represent a highly significant group of immune cells that form the foundation of immune defense enabling the body to fend off diverse infections and pathogens. The precise mechanisms underlying the role of Naive B cells in the development of gastrointestinal tumors remain incompletely explained. However, this study has partially illuminated their crucial role in tumor occurrence, through the application of MR analysis.

In recent years, there has been a surge of interest in exploring the development of anti-cancer drugs targeting immune cells. Research has demonstrated that immune infiltration within the tumor microenvironment plays a critical role in the development and progression of cancer, ultimately affecting clinical outcomes in cancer patients (47). Several immunotherapies, including adoptive cell transfer (ACT) and immune checkpoint inhibitors (ICIs), have achieved persistent clinical responses, yet their efficacy varies and only a subset of cancer patients benefits from them (48–51). Therefore, a comprehensive analysis of the immune cells infiltrating the tumor will help elucidate the mechanisms underlying tumor immune evasion, ultimately providing opportunities for the development of novel therapeutic strategies (51–53). This study utilized MR analysis to screen and identify immune cells causally linked to gastrointestinal tract cancers, therefore providing novel potential therapeutic targets for immunotherapy and informing the development of targeted prevention and treatment strategies.

Our study has some limitations that should be taken into careful consideration. Firstly, despite conducting multiple sensitivity analyses, it was challenging to fully assess the extent of multiple horizontal pleiotropy. Additionally, due to the lack of access to more detailed clinical information for all individuals included in the analysis, we were unable to perform further stratified analyses on the study population. Furthermore, since our MR analysis was based on publicly available databases of European ancestry, the generalizability of our findings to other populations needs to be treated with caution. Then, in order to comprehensively describe, identify, and screen immune traits causally linked to gastrointestinal tract cancers, we used relatively lenient thresholds to assess our findings, which may increase the risk of false positives. Finally, our MR analysis results did not identify immune traits causally linked to the onset of gastrointestinal tumors across all sites, which may indirectly suggest substantial heterogeneity among gastrointestinal tumors at different locations and considerable divergence in immune factors associated with their onset. Nevertheless, our study comprehensively evaluated the causal relationships between various immune traits and the onset of gastrointestinal tumors at different sites, providing valuable resources and insights for further exploration of future immunotherapeutic strategies.

## 5 Conclusions

In conclusion, our comprehensive MR analysis has furnished substantiation for the existence of causal links between diverse immune traits and gastrointestinal tract cancers. This revelation not only expands the horizons for investigators delving into the intricate biological underpinnings of gastrointestinal tract cancers but also plays a pivotal role in advancing our understanding of strategies for prevention and management in this context.

## Data availability statement

The original contributions presented in the study are included in the article/**Supplementary Material**. Further inquiries can be directed to the corresponding authors.

## Ethics statement

The availability of large-scale genomic data from publicly accessible databases, such as the FinnGen and GWAS Catalog, has facilitated the use of such data in genetic epidemiology research. As the data used in our study were obtained from these publicly accessible databases and do not contain any identifiable information, it was deemed unnecessary for approval from a medical ethics committee board. Our study adhered to all relevant laws and regulations regarding the use of human genetic data, and we aim to promote open access of genetic data to facilitate scientific discoveries in the field of genetics.

## Author contributions

Y-XW: Conceptualization, Data curation, Formal analysis, Funding acquisition, Writing – original draft. C-PZ: Investigation, Methodology, Project administration, Resources, Writing – review & editing. D-TW: Software, Supervision, Validation, Visualization, Writing – review & editing. JM: Conceptualization, Investigation, Software, Writing – review & editing. X-HS: Data curation, Methodology, Supervision, Writing – review & editing. YW: Formal analysis, Project administration, Validation, Writing – original draft. Y-MZ: Funding acquisition, Resources, Visualization, Writing – review & editing.

## Supplementary material

Supplementary Figure 1 The immune traits with *p-*values less than 0.05 obtained by the IVW method in esophageal cancer **(A)**, gastric cancer **(B)**, small intestine cancer **(C)**, colon cancer **(D)**, and rectal cancer **(E)**.

Supplementary Figure 2 The funnel plot **(A)**, scatter plot **(B)**, and leave-one-out sensitivity analysis for three immune traits with the maximum and minimum ORs associated with esophagus cancer.

Supplementary Figure 3 The funnel plot **(A)**, scatter plot **(B)**, and leave-one-out sensitivity analysis for three immune traits with the maximum and minimum ORs associated with stomach cancer.

Supplementary Figure 4 The funnel plot **(A)**, scatter plot **(B)**, and leave-one-out sensitivity analysis for three immune traits with the maximum and minimum ORs associated with cancer of small intestine.

Supplementary Figure 5 The funnel plot **(A)**, scatter plot **(B)**, and leave-one-out sensitivity analysis for three immune traits with the maximum and minimum ORs associated with colon cancer.

Supplementary Figure 6 The funnel plot **(A)**, scatter plot **(B)**, and leave-one-out sensitivity analysis for three immune traits with the maximum and minimum ORs associated with rectal cancer.

Supplementary Table 1 The information of 731 immunophenotypes in the GWAS Catalog.

Supplementary Table 2 The number of patients, and the URL for downloading the data for each type of gastrointestinal tract cancer.

Supplementary Table 3 The IVs associated with different immune traits.

Supplementary Table 4 The validation results from the BBJ database.
